# [4,6-Dimethyl­pyrimidine-2(1*H*)-thione-κ*S*]iodidobis(triphenyl­phosphane-κ*P*)copper(I)

**DOI:** 10.1107/S1600536812021010

**Published:** 2012-05-16

**Authors:** Chaveng Pakawatchai, Yupa Wattanakanjana, Patcharanan Choto, Ruthairat Nimthong

**Affiliations:** a Department of Chemistry and Center for Innovation in Chemistry, Faculty of Science, Prince of Songkla University, Hat Yai, Songkhla 90112, Thailand; b Department of Chemistry, Faculty of Science, Prince of Songkla University, Hat Yai 90112, Thailand

## Abstract

In the mononuclear title complex, [CuI(C_6_H_8_N_2_S)(C_18_H_15_P)_2_], the Cu^I^ ion is in a slightly distorted tetra­hedral coordination geometry formed by two P atoms from two triphenyl­phosphane ligands, one S atom from a 4,6-dimethyl­pyrimidine-2(1*H*)-thione ligand and one iodide ion. There is an intra­molecular N—H⋯I hydrogen bond. In the crystal, π–π stacking inter­actions [centroid–centroid distance = 3.594 (1) Å] are observed.

## Related literature

For the coordination and potential applications of Cu^I^ complexes, see: Santra *et al.* (1999 ▶); Fujisawa *et al.* (2004 ▶); Tian *et al.* (2004 ▶); Kang (2006 ▶); Reymond & Cossy (2008 ▶); Gong *et al.* (2010 ▶). For relevant examples of discrete complexes, see: Voutsas *et al.* (1995 ▶); Lemos *et al.* (2001 ▶); Lobana *et al.* (2008 ▶); Nimthong *et al.* (2008 ▶).
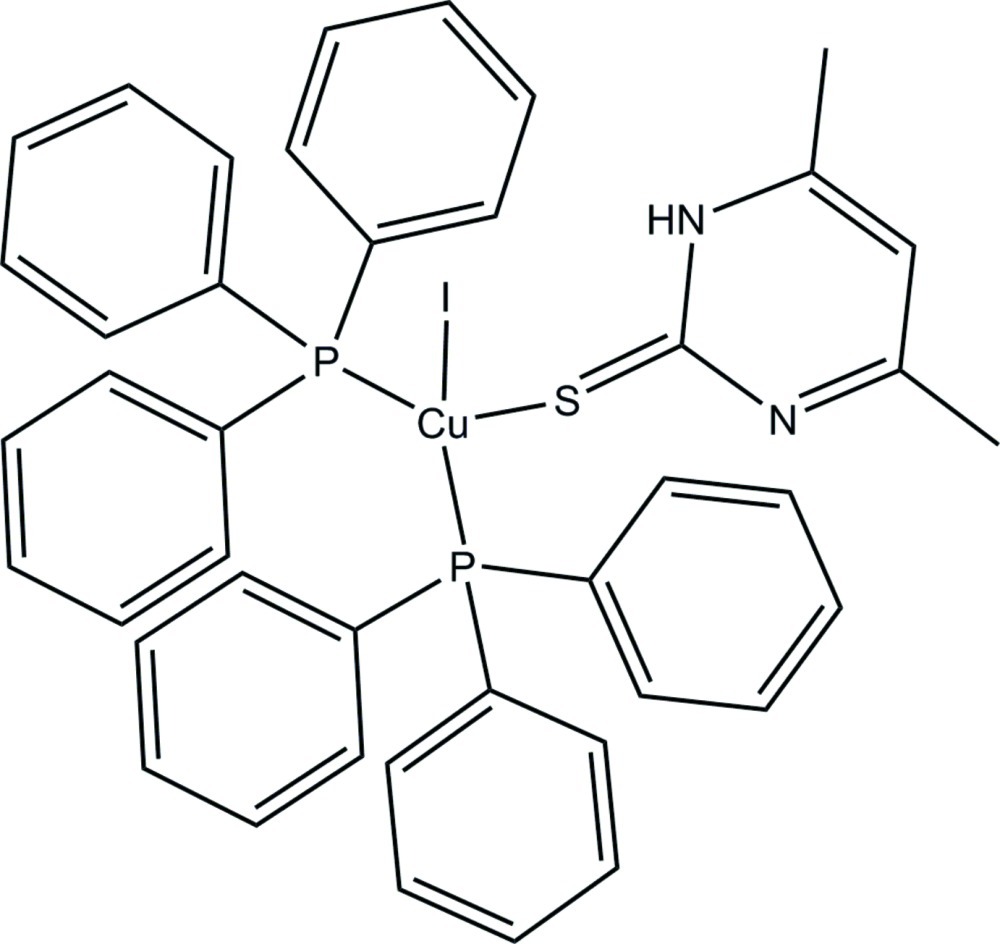


## Experimental

### 

#### Crystal data

[CuI(C_6_H_8_N_2_S)(C_18_H_15_P)_2_]
*M* *_r_* = 855.18
Triclinic, 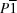

*a* = 11.5605 (7) Å
*b* = 13.0076 (8) Å
*c* = 13.6456 (8) Å
α = 92.243 (1)°
β = 99.247 (1)°
γ = 106.092 (1)°
*V* = 1938.3 (2) Å^3^
*Z* = 2
Mo *K*α radiation
μ = 1.53 mm^−1^
*T* = 293 K
0.32 × 0.16 × 0.08 mm

#### Data collection

Bruker SMART CCD diffractometer
Absorption correction: multi-scan (*SADABS*; Bruker, 2003 ▶) *T*_min_ = 0.744, *T*_max_ = 0.882
26730 measured reflections
9368 independent reflections
8066 reflections with *I* > 2σ(*I*)
*R*_int_ = 0.019

#### Refinement

*R*[*F* ^2^ > 2σ(*F* ^2^)] = 0.028
*wR*(*F* ^2^) = 0.075
*S* = 1.03
9368 reflections
448 parameters
H atoms treated by a mixture of independent and constrained refinement
Δρ_max_ = 0.90 e Å^−3^
Δρ_min_ = −0.26 e Å^−3^

### 

Data collection: *SMART* (Bruker, 1998 ▶); cell refinement: *SAINT* (Bruker, 2003 ▶); data reduction: *SAINT*; program(s) used to solve structure: *SHELXS97* (Sheldrick, 2008) ▶; program(s) used to refine structure: *SHELXL97* (Sheldrick, 2008) ▶; molecular graphics: *Mercury* (Macrae *et al.*, 2008 ▶); software used to prepare material for publication: *SHELXL97* and *publCIF* (Westrip, 2010 ▶).

## Supplementary Material

Crystal structure: contains datablock(s) I, global. DOI: 10.1107/S1600536812021010/lh5472sup1.cif

Structure factors: contains datablock(s) I. DOI: 10.1107/S1600536812021010/lh5472Isup2.hkl

Additional supplementary materials:  crystallographic information; 3D view; checkCIF report

## Figures and Tables

**Table 1:** Hydrogen-bond geometry (Å, °)

*D*—H⋯*A*	*D*—H	H⋯*A*	*D*⋯*A*	*D*—H⋯*A*
N1—H1*N*⋯I1	0.87 (3)	2.62 (3)	3.4858 (18)	176 (3)

**Fig. 1.**
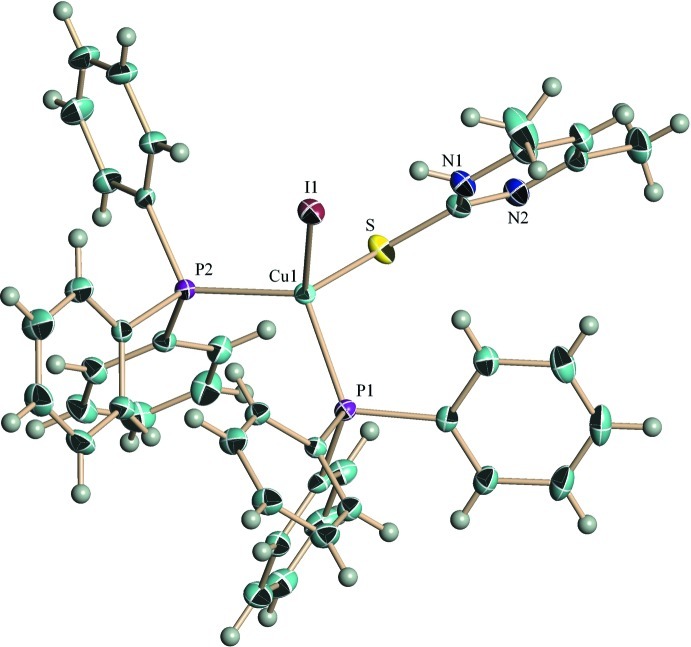
The molecular structure with displacement ellipsoids drawn at the 30% probability level.

**Fig. 2.**
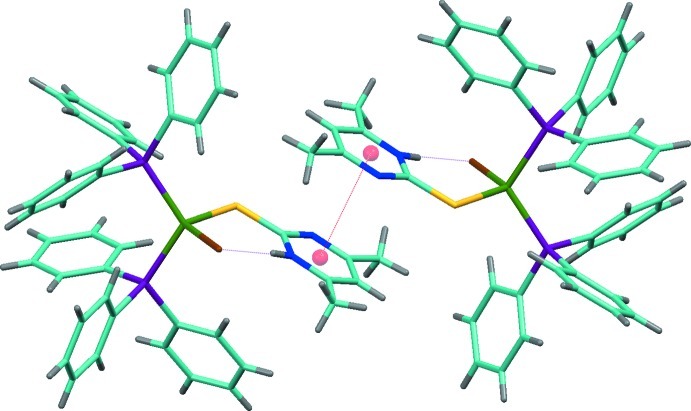
Part of the crystal structure with the intramolecular hydrogen bond and π–π stacking interactions shown asphosphine dashed lines.

## supplementary crystallographic information

### Comment

The synthesis and coordination chemistry of copper(I) complexes have been widely studied. Some of these complexes have been found to have unusual structural features, exhibit corrosion-inhibiting properties (Tian *et al.*, 2004), catalytic activity in photo-redox reactions (Santra *et al.*, 1999), phosphorescence due to close Cu···Cu interactions (Gong, *et al.*, 2010), precursors to blue copper–protein model (Fujisawa *et al*., 2004) and catalysts in enantiomer selective Diels–Alder reactions (Reymond & Cossy, 2008). Moreover, the role of copper(I) is evident in several biologically important reactions, such as a dioxygen carrier and models for several enzymes (Kang, 2006).

The molecular structure of the title compound is shown in Fig. 1. The complex is monomeric with a slightly distorted tetrahedral coordination enviroment around Cu1. The Cu1—S bond length of 2.3404 (6) Å, is in good agreement with values reported for other copper(I) complexes with heterocyclic thione ligands, such as 2.3723 (12) Å for [Cu(N_3_)(dmpymtH)(PPh_3_)_2_] (Lemos *et al.*, 2001) and 2.344 (3) Å for [CuI(1κs-imzsH)(PPh_3_)_2_] (Lobana *et al.*, 2008). The Cu1—P1 and Cu1—P2 bond distances, 2.2897 (5) and 2.3047 (5) Å, are as expected. The bond distance of Cu1—I1, 2.6801 (3), is comparable to those found [2.6658 (8) Å] for [Cu_2_(C_7_H_8_N_2_S)(C_18_ H_13_P)_2_I] (Nimthong *et al.*, 2008) and 2.674 (2) Å for [Cu(PPh_3_)_2_(pymtH)I] (Voutsas *et al.*, 1995). In the crystal, π(pyrimidine)···π(pyrimidine) (centroid-centroid distances = 3.594 Å) interactions are observed. In addition, an intramolecular hydrogen bond is also observed (see Table 1 and Fig. 2).

### Experimental

A solution of 4,6-dimethylpyrimidine-2(1*H*)-thione, (0.08 g, 0.52 mmol) in 30 cm^3^ of methanol was stirred at 333 K then CuI (0.10 g, 0.52 mmol) solid was added and stirred for 3 h. Solid of triphenylphosphane (0.27 g, 1.04 mmol) was added and heated with continuous stirring for a period of 2 h. The clear yellow solution was formed then filtered off and kept at room temperature. Slow evaporation of the solvent gave the yellow colored crystalline solids, which were filtered off and dried *in vacuo*. Analysis found: C 60.11, H 4.46, N 2.88, S 3.22%; calculated for C_42_H_37_CuIN_2_P_2_S: C 59.03, H 4.37, N 3.28, S 3.76%.

### Refinement

The H atoms bonded to C atoms were constrained with a riding model of 0.93–0.96 Å, and *U*_iso_(H) = 1.2*U*_eq_(C). The H atom bonded to the N atom was located in a difference Fourier map and refined isotropically.

### Crystal data

**Table:**

[CuI(C_6_H_8_N_2_S)(C_18_H_15_P)_2_]	*Z* = 2
*M**_r_* = 855.18	*F*(000) = 864
Triclinic, *P*1	*D*_x_ = 1.465 Mg m^−^^3^
Hall symbol: -P 1	Mo *K*α radiation, λ = 0.71073 Å
*a* = 11.5605 (7) Å	Cell parameters from 9368 reflections
*b* = 13.0076 (8) Å	θ = 1.5–28.1°
*c* = 13.6456 (8) Å	µ = 1.53 mm^−^^1^
α = 92.243 (1)°	*T* = 293 K
β = 99.247 (1)°	Block, yellow
γ = 106.092 (1)°	0.32 × 0.16 × 0.08 mm
*V* = 1938.3 (2) Å^3^	

### Data collection

**Table:**

Bruker SMART CCD diffractometer	9368 independent reflections
Radiation source: fine-focus sealed tube	8066 reflections with *I* > 2σ(*I*)
Graphite monochromator	*R*_int_ = 0.019
φ and ω scans	θ_max_ = 28.1°, θ_min_ = 1.5°
Absorption correction: multi-scan (*SADABS*; Bruker, 2003)	*h* = −15→15
*T*_min_ = 0.744, *T*_max_ = 0.882	*k* = −17→17
26730 measured reflections	*l* = −18→18

### Refinement

**Table:**

Refinement on *F*^2^	Primary atom site location: structure-invariant direct methods
Least-squares matrix: full	Secondary atom site location: difference Fourier map
*R*[*F*^2^ > 2σ(*F*^2^)] = 0.028	Hydrogen site location: inferred from neighbouring sites
*wR*(*F*^2^) = 0.075	H atoms treated by a mixture of independent and constrained refinement
*S* = 1.03	*w* = 1/[σ^2^(*F*_o_^2^) + (0.040*P*)^2^ + 0.4017*P*] where *P* = (*F*_o_^2^ + 2*F*_c_^2^)/3
9368 reflections	(Δ/σ)_max_ = 0.009
448 parameters	Δρ_max_ = 0.90 e Å^−^^3^
0 restraints	Δρ_min_ = −0.26 e Å^−^^3^

### Special details

**Table:**

Geometry. All e.s.d.'s (except the e.s.d. in the dihedral angle between two l.s. planes) are estimated using the full covariance matrix. The cell e.s.d.'s are taken into account individually in the estimation of e.s.d.'s in distances, angles and torsion angles; correlations between e.s.d.'s in cell parameters are only used when they are defined by crystal symmetry. An approximate (isotropic) treatment of cell e.s.d.'s is used for estimating e.s.d.'s involving l.s. planes.
Refinement. Refinement of *F*^2^ against ALL reflections. The weighted *R*-factor *wR* and goodness of fit *S* are based on *F*^2^, conventional *R*-factors *R* are based on *F*, with *F* set to zero for negative *F*^2^. The threshold expression of *F*^2^ > σ(*F*^2^) is used only for calculating *R*-factors(gt) *etc*. and is not relevant to the choice of reflections for refinement. *R*-factors based on *F*^2^ are statistically about twice as large as those based on *F*, and *R*- factors based on ALL data will be even larger.

### Fractional atomic coordinates and isotropic or equivalent isotropic displacement parameters (Å^2^)

**Table:**

	*x*	*y*	*z*	*U*_iso_*/*U*_eq_	
C28	0.1739 (3)	0.7348 (2)	0.7628 (3)	0.0828 (9)	
H28	0.1874	0.8068	0.7511	0.099*	
N1	0.30622 (16)	0.47879 (16)	0.54816 (13)	0.0497 (4)	
N2	0.50121 (16)	0.58089 (15)	0.62532 (14)	0.0522 (4)	
I1	0.059857 (12)	0.249292 (11)	0.536351 (9)	0.05129 (5)	
Cu1	0.20767 (2)	0.292271 (17)	0.714703 (16)	0.03664 (6)	
P1	0.23312 (4)	0.13349 (4)	0.76683 (3)	0.03493 (10)	
P2	0.11163 (4)	0.37674 (4)	0.81382 (3)	0.03521 (10)	
S	0.40255 (5)	0.40670 (5)	0.71098 (4)	0.05366 (14)	
C25	0.13677 (17)	0.52001 (15)	0.79783 (16)	0.0419 (4)	
C36	0.2937 (2)	0.4210 (2)	0.97987 (18)	0.0589 (6)	
H36	0.3434	0.4507	0.9349	0.071*	
C31	0.16908 (18)	0.37741 (15)	0.94672 (14)	0.0413 (4)	
C7	0.08651 (17)	0.04295 (14)	0.78284 (14)	0.0390 (4)	
C13	0.33044 (18)	0.13894 (16)	0.88752 (14)	0.0413 (4)	
C20	−0.12265 (19)	0.38591 (17)	0.84239 (16)	0.0479 (5)	
H20	−0.0832	0.4533	0.8758	0.057*	
C19	−0.05481 (16)	0.32710 (14)	0.80249 (13)	0.0361 (4)	
C1A	0.40371 (17)	0.49478 (16)	0.62299 (15)	0.0436 (4)	
C15	0.3912 (3)	0.0614 (3)	1.03790 (19)	0.0688 (7)	
H15	0.3791	0.0047	1.0782	0.083*	
C1	0.29216 (18)	0.05372 (14)	0.68461 (15)	0.0413 (4)	
C24	−0.11585 (18)	0.22715 (16)	0.75210 (15)	0.0457 (4)	
H24	−0.0721	0.1869	0.7244	0.055*	
C12	0.0372 (2)	0.06523 (18)	0.86484 (17)	0.0519 (5)	
H12	0.0823	0.1209	0.9125	0.062*	
C23	−0.2420 (2)	0.18691 (19)	0.74285 (18)	0.0582 (6)	
H23	−0.2822	0.1197	0.7093	0.070*	
C30	0.1607 (2)	0.59860 (18)	0.8761 (2)	0.0595 (6)	
H30	0.1647	0.5793	0.9412	0.071*	
C4A	0.4968 (2)	0.64843 (17)	0.55446 (18)	0.0528 (5)	
C3A	0.3966 (2)	0.63329 (19)	0.47959 (19)	0.0590 (6)	
H3A	0.3960	0.6827	0.4322	0.071*	
C21	−0.2480 (2)	0.3452 (2)	0.83287 (19)	0.0588 (6)	
H21	−0.2924	0.3850	0.8603	0.071*	
C26	0.1312 (2)	0.5507 (2)	0.70146 (18)	0.0577 (5)	
H26	0.1153	0.4991	0.6481	0.069*	
C6	0.3865 (2)	0.0101 (2)	0.7182 (2)	0.0607 (6)	
H6	0.4233	0.0216	0.7850	0.073*	
C14	0.3145 (2)	0.0530 (2)	0.94765 (17)	0.0549 (5)	
H14	0.2518	−0.0100	0.9266	0.066*	
C11	−0.0780 (2)	0.0054 (2)	0.8761 (2)	0.0636 (6)	
H11	−0.1099	0.0205	0.9316	0.076*	
C8	0.0176 (2)	−0.03985 (19)	0.71278 (18)	0.0577 (6)	
H8	0.0489	−0.0563	0.6575	0.069*	
C18	0.4263 (2)	0.23035 (18)	0.91961 (17)	0.0545 (5)	
H18	0.4394	0.2878	0.8803	0.065*	
C32	0.0970 (2)	0.33340 (18)	1.01459 (16)	0.0523 (5)	
H32	0.0132	0.3033	0.9940	0.063*	
C22	−0.3075 (2)	0.2458 (2)	0.78291 (19)	0.0608 (6)	
H22	−0.3920	0.2186	0.7764	0.073*	
C10	−0.1458 (2)	−0.0765 (2)	0.8056 (2)	0.0730 (7)	
H10	−0.2236	−0.1167	0.8130	0.088*	
C35	0.3454 (3)	0.4211 (3)	1.0784 (2)	0.0741 (8)	
H35	0.4292	0.4510	1.0994	0.089*	
C2	0.2401 (2)	0.03643 (19)	0.58387 (17)	0.0567 (5)	
H2	0.1786	0.0671	0.5598	0.068*	
C2A	0.2996 (2)	0.5461 (2)	0.47577 (18)	0.0589 (6)	
C17	0.5035 (3)	0.2368 (2)	1.0107 (2)	0.0738 (8)	
H17	0.5676	0.2988	1.0318	0.089*	
C16	0.4861 (3)	0.1539 (3)	1.0685 (2)	0.0743 (8)	
H16	0.5382	0.1592	1.1292	0.089*	
C6A	0.6067 (3)	0.7428 (2)	0.5611 (3)	0.0796 (8)	
H6A1	0.6128	0.7903	0.6185	0.119*	
H6A2	0.5994	0.7801	0.5021	0.119*	
H6A3	0.6787	0.7187	0.5668	0.119*	
C33	0.1498 (3)	0.3341 (2)	1.11417 (18)	0.0693 (7)	
H33	0.1009	0.3051	1.1599	0.083*	
C34	0.2736 (3)	0.3774 (2)	1.1448 (2)	0.0761 (8)	
H34	0.3085	0.3770	1.2111	0.091*	
C5A	0.1858 (3)	0.5182 (3)	0.3989 (2)	0.1030 (13)	
H5A1	0.1244	0.5432	0.4238	0.155*	
H5A2	0.1567	0.4417	0.3840	0.155*	
H5A3	0.2032	0.5519	0.3395	0.155*	
C5	0.4258 (3)	−0.0509 (3)	0.6519 (3)	0.0819 (9)	
H5	0.4898	−0.0793	0.6744	0.098*	
C3	0.2792 (3)	−0.0261 (2)	0.5190 (2)	0.0716 (7)	
H3	0.2428	−0.0384	0.4520	0.086*	
C4	0.3712 (3)	−0.0695 (2)	0.5536 (2)	0.0793 (9)	
H4	0.3969	−0.1119	0.5101	0.095*	
C29	0.1787 (3)	0.7050 (2)	0.8583 (3)	0.0758 (8)	
H29	0.1942	0.7569	0.9114	0.091*	
C9	−0.0984 (2)	−0.0983 (2)	0.7250 (2)	0.0791 (8)	
H9	−0.1446	−0.1534	0.6772	0.095*	
C27	0.1490 (3)	0.6578 (2)	0.6843 (2)	0.0777 (8)	
H27	0.1442	0.6777	0.6194	0.093*	
H1N	0.247 (3)	0.420 (2)	0.547 (2)	0.078 (9)*	

### Atomic displacement parameters (Å^2^)

**Table:**

	*U*^11^	*U*^22^	*U*^33^	*U*^12^	*U*^13^	*U*^23^
C28	0.0644 (16)	0.0417 (13)	0.141 (3)	0.0128 (12)	0.0139 (18)	0.0234 (17)
N1	0.0387 (9)	0.0553 (11)	0.0455 (9)	0.0002 (8)	0.0018 (7)	0.0137 (8)
N2	0.0393 (9)	0.0523 (10)	0.0570 (11)	0.0014 (8)	0.0059 (8)	0.0074 (8)
I1	0.04691 (8)	0.05672 (9)	0.03803 (8)	0.00194 (6)	−0.00378 (5)	−0.00007 (6)
Cu1	0.03788 (12)	0.03647 (12)	0.03407 (11)	0.00853 (9)	0.00599 (9)	0.00272 (9)
P1	0.0365 (2)	0.0340 (2)	0.0347 (2)	0.00952 (18)	0.00871 (18)	0.00224 (18)
P2	0.0338 (2)	0.0356 (2)	0.0343 (2)	0.00938 (18)	0.00254 (18)	−0.00113 (18)
S	0.0366 (2)	0.0665 (3)	0.0475 (3)	0.0004 (2)	−0.0008 (2)	0.0205 (2)
C25	0.0321 (9)	0.0378 (9)	0.0543 (11)	0.0091 (7)	0.0050 (8)	0.0045 (8)
C36	0.0478 (12)	0.0735 (15)	0.0511 (13)	0.0197 (11)	−0.0040 (10)	−0.0108 (11)
C31	0.0464 (10)	0.0423 (10)	0.0352 (9)	0.0187 (8)	−0.0011 (8)	−0.0062 (7)
C7	0.0376 (9)	0.0364 (9)	0.0447 (10)	0.0111 (7)	0.0105 (8)	0.0078 (8)
C13	0.0419 (10)	0.0473 (10)	0.0392 (10)	0.0198 (8)	0.0076 (8)	0.0049 (8)
C20	0.0456 (11)	0.0457 (11)	0.0520 (12)	0.0134 (9)	0.0090 (9)	−0.0031 (9)
C19	0.0342 (9)	0.0399 (9)	0.0339 (9)	0.0105 (7)	0.0051 (7)	0.0033 (7)
C1A	0.0340 (9)	0.0501 (11)	0.0424 (10)	0.0052 (8)	0.0064 (8)	0.0063 (8)
C15	0.0698 (16)	0.093 (2)	0.0561 (14)	0.0397 (15)	0.0136 (12)	0.0321 (14)
C1	0.0449 (10)	0.0329 (9)	0.0487 (11)	0.0086 (8)	0.0206 (9)	0.0018 (8)
C24	0.0410 (10)	0.0466 (11)	0.0474 (11)	0.0099 (8)	0.0085 (8)	−0.0046 (8)
C12	0.0540 (12)	0.0546 (12)	0.0484 (12)	0.0127 (10)	0.0188 (10)	0.0042 (9)
C23	0.0449 (11)	0.0560 (13)	0.0625 (14)	−0.0012 (10)	0.0088 (10)	−0.0096 (11)
C30	0.0603 (14)	0.0433 (11)	0.0696 (15)	0.0089 (10)	0.0095 (12)	−0.0041 (10)
C4A	0.0460 (11)	0.0459 (11)	0.0656 (14)	0.0075 (9)	0.0174 (10)	0.0075 (10)
C3A	0.0579 (13)	0.0559 (13)	0.0653 (15)	0.0139 (11)	0.0169 (11)	0.0248 (11)
C21	0.0489 (12)	0.0665 (14)	0.0676 (15)	0.0228 (11)	0.0197 (11)	−0.0006 (12)
C26	0.0566 (13)	0.0569 (13)	0.0584 (13)	0.0179 (11)	0.0021 (11)	0.0128 (11)
C6	0.0605 (14)	0.0649 (15)	0.0646 (15)	0.0274 (12)	0.0188 (12)	0.0008 (12)
C14	0.0509 (12)	0.0621 (13)	0.0546 (13)	0.0191 (11)	0.0096 (10)	0.0175 (10)
C11	0.0568 (14)	0.0752 (16)	0.0663 (15)	0.0175 (12)	0.0318 (12)	0.0194 (13)
C8	0.0476 (12)	0.0582 (13)	0.0604 (14)	0.0034 (10)	0.0135 (10)	−0.0086 (11)
C18	0.0553 (13)	0.0501 (12)	0.0539 (13)	0.0164 (10)	−0.0042 (10)	0.0009 (10)
C32	0.0597 (13)	0.0524 (12)	0.0429 (11)	0.0169 (10)	0.0025 (9)	0.0044 (9)
C22	0.0376 (11)	0.0735 (16)	0.0674 (15)	0.0082 (10)	0.0136 (10)	0.0014 (12)
C10	0.0452 (13)	0.0726 (17)	0.097 (2)	0.0019 (12)	0.0246 (14)	0.0171 (15)
C35	0.0616 (15)	0.096 (2)	0.0607 (16)	0.0371 (15)	−0.0184 (13)	−0.0229 (14)
C2	0.0665 (14)	0.0596 (13)	0.0490 (12)	0.0225 (11)	0.0183 (11)	−0.0018 (10)
C2A	0.0535 (13)	0.0669 (15)	0.0515 (13)	0.0109 (11)	0.0041 (10)	0.0193 (11)
C17	0.0651 (16)	0.0727 (17)	0.0712 (17)	0.0201 (13)	−0.0208 (13)	−0.0064 (14)
C16	0.0705 (17)	0.103 (2)	0.0530 (14)	0.0422 (17)	−0.0090 (12)	0.0069 (14)
C6A	0.0615 (16)	0.0562 (15)	0.110 (2)	−0.0056 (12)	0.0211 (16)	0.0166 (15)
C33	0.095 (2)	0.0756 (17)	0.0410 (12)	0.0339 (15)	0.0062 (12)	0.0104 (11)
C34	0.099 (2)	0.089 (2)	0.0442 (13)	0.0519 (18)	−0.0173 (14)	−0.0088 (13)
C5A	0.0741 (19)	0.124 (3)	0.082 (2)	−0.0040 (19)	−0.0207 (16)	0.055 (2)
C5	0.083 (2)	0.085 (2)	0.099 (2)	0.0493 (17)	0.0372 (18)	0.0027 (17)
C3	0.0882 (19)	0.0708 (16)	0.0584 (15)	0.0197 (15)	0.0300 (14)	−0.0112 (12)
C4	0.094 (2)	0.0695 (17)	0.086 (2)	0.0278 (16)	0.0473 (18)	−0.0101 (15)
C29	0.0727 (17)	0.0385 (12)	0.110 (2)	0.0092 (12)	0.0121 (16)	−0.0065 (14)
C9	0.0520 (14)	0.0715 (17)	0.095 (2)	−0.0115 (12)	0.0176 (14)	−0.0172 (15)
C27	0.0728 (18)	0.0692 (18)	0.092 (2)	0.0231 (14)	0.0062 (15)	0.0383 (16)

### Geometric parameters (Å, º)

**Table:**

C28—C29	1.372 (5)	C4A—C6A	1.490 (3)
C28—C27	1.375 (5)	C3A—C2A	1.349 (3)
C28—H28	0.9300	C3A—H3A	0.9300
N1—C2A	1.352 (3)	C21—C22	1.377 (3)
N1—C1A	1.356 (3)	C21—H21	0.9300
N1—H1N	0.87 (3)	C26—C27	1.386 (3)
N2—C4A	1.336 (3)	C26—H26	0.9300
N2—C1A	1.346 (2)	C6—C5	1.390 (3)
I1—Cu1	2.6801 (3)	C6—H6	0.9300
Cu1—P1	2.2897 (5)	C14—H14	0.9300
Cu1—P2	2.3047 (5)	C11—C10	1.374 (4)
Cu1—S	2.3404 (6)	C11—H11	0.9300
P1—C13	1.826 (2)	C8—C9	1.385 (3)
P1—C7	1.8276 (19)	C8—H8	0.9300
P1—C1	1.8301 (18)	C18—C17	1.394 (3)
P2—C31	1.8271 (19)	C18—H18	0.9300
P2—C19	1.8306 (18)	C32—C33	1.396 (3)
P2—C25	1.832 (2)	C32—H32	0.9300
S—C1A	1.691 (2)	C22—H22	0.9300
C25—C26	1.386 (3)	C10—C9	1.359 (4)
C25—C30	1.388 (3)	C10—H10	0.9300
C36—C35	1.380 (3)	C35—C34	1.362 (4)
C36—C31	1.385 (3)	C35—H35	0.9300
C36—H36	0.9300	C2—C3	1.388 (3)
C31—C32	1.380 (3)	C2—H2	0.9300
C7—C8	1.381 (3)	C2A—C5A	1.491 (4)
C7—C12	1.390 (3)	C17—C16	1.350 (4)
C13—C18	1.381 (3)	C17—H17	0.9300
C13—C14	1.401 (3)	C16—H16	0.9300
C20—C21	1.380 (3)	C6A—H6A1	0.9600
C20—C19	1.391 (3)	C6A—H6A2	0.9600
C20—H20	0.9300	C6A—H6A3	0.9600
C19—C24	1.388 (3)	C33—C34	1.372 (4)
C15—C14	1.378 (3)	C33—H33	0.9300
C15—C16	1.382 (4)	C34—H34	0.9300
C15—H15	0.9300	C5A—H5A1	0.9600
C1—C6	1.387 (3)	C5A—H5A2	0.9600
C1—C2	1.392 (3)	C5A—H5A3	0.9600
C24—C23	1.389 (3)	C5—C4	1.369 (5)
C24—H24	0.9300	C5—H5	0.9300
C12—C11	1.380 (3)	C3—C4	1.365 (4)
C12—H12	0.9300	C3—H3	0.9300
C23—C22	1.372 (3)	C4—H4	0.9300
C23—H23	0.9300	C29—H29	0.9300
C30—C29	1.378 (3)	C9—H9	0.9300
C30—H30	0.9300	C27—H27	0.9300
C4A—C3A	1.379 (3)		
			
C29—C28—C27	119.7 (2)	C25—C26—H26	119.8
C29—C28—H28	120.1	C27—C26—H26	119.8
C27—C28—H28	120.1	C1—C6—C5	120.0 (3)
C2A—N1—C1A	123.70 (19)	C1—C6—H6	120.0
C2A—N1—H1N	119.8 (19)	C5—C6—H6	120.0
C1A—N1—H1N	116.5 (19)	C15—C14—C13	120.3 (2)
C4A—N2—C1A	118.34 (19)	C15—C14—H14	119.8
P1—Cu1—P2	114.845 (19)	C13—C14—H14	119.8
P1—Cu1—S	107.14 (2)	C10—C11—C12	120.1 (2)
P2—Cu1—S	108.80 (2)	C10—C11—H11	119.9
P1—Cu1—I1	107.867 (15)	C12—C11—H11	119.9
P2—Cu1—I1	104.908 (15)	C7—C8—C9	119.9 (2)
S—Cu1—I1	113.453 (16)	C7—C8—H8	120.1
C13—P1—C7	102.91 (9)	C9—C8—H8	120.1
C13—P1—C1	102.99 (9)	C13—C18—C17	120.3 (2)
C7—P1—C1	104.14 (9)	C13—C18—H18	119.9
C13—P1—Cu1	117.33 (7)	C17—C18—H18	119.9
C7—P1—Cu1	110.26 (6)	C31—C32—C33	120.1 (2)
C1—P1—Cu1	117.49 (6)	C31—C32—H32	120.0
C31—P2—C19	104.15 (9)	C33—C32—H32	120.0
C31—P2—C25	102.49 (9)	C23—C22—C21	119.9 (2)
C19—P2—C25	102.70 (8)	C23—C22—H22	120.0
C31—P2—Cu1	112.72 (6)	C21—C22—H22	120.0
C19—P2—Cu1	118.86 (6)	C9—C10—C11	119.6 (2)
C25—P2—Cu1	114.09 (7)	C9—C10—H10	120.2
C1A—S—Cu1	113.57 (7)	C11—C10—H10	120.2
C26—C25—C30	118.6 (2)	C34—C35—C36	119.9 (3)
C26—C25—P2	117.47 (17)	C34—C35—H35	120.1
C30—C25—P2	123.89 (17)	C36—C35—H35	120.1
C35—C36—C31	121.2 (3)	C3—C2—C1	120.6 (2)
C35—C36—H36	119.4	C3—C2—H2	119.7
C31—C36—H36	119.4	C1—C2—H2	119.7
C32—C31—C36	118.4 (2)	C3A—C2A—N1	117.0 (2)
C32—C31—P2	124.09 (16)	C3A—C2A—C5A	125.5 (2)
C36—C31—P2	117.43 (17)	N1—C2A—C5A	117.5 (2)
C8—C7—C12	118.64 (19)	C16—C17—C18	120.6 (3)
C8—C7—P1	123.06 (15)	C16—C17—H17	119.7
C12—C7—P1	118.01 (15)	C18—C17—H17	119.7
C18—C13—C14	118.5 (2)	C17—C16—C15	120.2 (2)
C18—C13—P1	118.66 (16)	C17—C16—H16	119.9
C14—C13—P1	122.82 (17)	C15—C16—H16	119.9
C21—C20—C19	120.6 (2)	C4A—C6A—H6A1	109.5
C21—C20—H20	119.7	C4A—C6A—H6A2	109.5
C19—C20—H20	119.7	H6A1—C6A—H6A2	109.5
C24—C19—C20	118.64 (18)	C4A—C6A—H6A3	109.5
C24—C19—P2	118.91 (14)	H6A1—C6A—H6A3	109.5
C20—C19—P2	122.45 (15)	H6A2—C6A—H6A3	109.5
N2—C1A—N1	119.00 (18)	C34—C33—C32	120.2 (3)
N2—C1A—S	120.84 (15)	C34—C33—H33	119.9
N1—C1A—S	120.16 (15)	C32—C33—H33	119.9
C14—C15—C16	120.0 (2)	C35—C34—C33	120.1 (2)
C14—C15—H15	120.0	C35—C34—H34	119.9
C16—C15—H15	120.0	C33—C34—H34	119.9
C6—C1—C2	118.65 (19)	C2A—C5A—H5A1	109.5
C6—C1—P1	122.95 (17)	C2A—C5A—H5A2	109.5
C2—C1—P1	118.40 (16)	H5A1—C5A—H5A2	109.5
C19—C24—C23	120.29 (19)	C2A—C5A—H5A3	109.5
C19—C24—H24	119.9	H5A1—C5A—H5A3	109.5
C23—C24—H24	119.9	H5A2—C5A—H5A3	109.5
C11—C12—C7	120.6 (2)	C4—C5—C6	120.5 (3)
C11—C12—H12	119.7	C4—C5—H5	119.7
C7—C12—H12	119.7	C6—C5—H5	119.7
C22—C23—C24	120.3 (2)	C4—C3—C2	119.9 (3)
C22—C23—H23	119.8	C4—C3—H3	120.1
C24—C23—H23	119.8	C2—C3—H3	120.1
C29—C30—C25	120.6 (3)	C3—C4—C5	120.3 (2)
C29—C30—H30	119.7	C3—C4—H4	119.8
C25—C30—H30	119.7	C5—C4—H4	119.8
N2—C4A—C3A	122.6 (2)	C28—C29—C30	120.4 (3)
N2—C4A—C6A	116.1 (2)	C28—C29—H29	119.8
C3A—C4A—C6A	121.3 (2)	C30—C29—H29	119.8
C2A—C3A—C4A	119.3 (2)	C10—C9—C8	121.2 (3)
C2A—C3A—H3A	120.3	C10—C9—H9	119.4
C4A—C3A—H3A	120.3	C8—C9—H9	119.4
C22—C21—C20	120.2 (2)	C28—C27—C26	120.3 (3)
C22—C21—H21	119.9	C28—C27—H27	119.8
C20—C21—H21	119.9	C26—C27—H27	119.8
C25—C26—C27	120.3 (3)		

### Hydrogen-bond geometry (Å, º)

**Table:**

*D*—H···*A*	*D*—H	H···*A*	*D*···*A*	*D*—H···*A*
N1—H1*N*···I1	0.87 (3)	2.62 (3)	3.4858 (18)	176 (3)
